# Considerations for Dose Selection and Clinical Pharmacokinetics/Pharmacodynamics for the Development of Antibacterial Agents

**DOI:** 10.1128/AAC.02309-18

**Published:** 2019-04-25

**Authors:** M. L. Rizk, S. M. Bhavnani, G. Drusano, A. Dane, A. E. Eakin, T. Guina, S. H. Jang, J. F. Tomayko, J. Wang, L. Zhuang, T. P. Lodise

**Affiliations:** a Merck & Co. Inc., Kenilworth, New Jersey, USA; b Institute for Clinical Pharmacodynamics, Inc., Schenectady, New York, USA; c Institute for Therapeutic Innovation, University of Florida, Orlando, Florida, USA; d DaneStat Consulting Limited, Macclesfield, United Kingdom; e National Institute of Allergy and Infectious Diseases, National Institutes of Health, Bethesda, Maryland, USA; f Office of Clinical Pharmacology, Office of Translational Sciences, U.S. Food and Drug Administration, Silver Spring, Maryland, USA; g Pfizer, Wellesley, Massachusetts, USA; h Albany College of Pharmacy and Health Sciences, Albany, New York, USA

**Keywords:** antibacterial, best practices/recommendations, clinical, dose selection, drug development, drug penetration, patient population, pharmacokinetics/pharmacodynamics, translation, workshop

## Abstract

In June 2017, The National Institute of Allergy and Infectious Diseases, part of the National Institutes of Health, organized a workshop entitled “Pharmacokinetics-Pharmacodynamics (PK/PD) for Development of Therapeutics against Bacterial Pathogens” to discuss details and critical parameters of various PK/PD methods and identify approaches for linking human pharmacokinetic (PK) data and drug efficacy analyses. The workshop participants included individuals from academia, industry, and government. This and the accompanying minireview on nonclinical PK/PD summarize the workshop discussions and recommendations. It is important to consider how information like PK/PD can support the clinical effectiveness of new antibacterial drugs, as PK/PD data have become central to antibacterial drug development programs. Key clinical considerations for antibacterial dose selection and clinical PK/PD characterization discussed in this minireview include a robust assessment of PK in the patient population of interest, critical considerations for assessing drug penetration in the lung for the treatment of pneumonia, and an emphasis on special populations, including patients with renal impairment and augmented renal function, as well as on dosing in obese and pediatric patients. Successful application of such approaches is now used to provide a more informative drug development package to support the approval of new antibiotics.

## Abstract (precis)

In June 2017, The National Institute of Allergy and Infectious Diseases, part of the National Institutes of Health, organized a workshop entitled “Pharmacokinetics-Pharmacodynamics (PK/PD) for Development of Therapeutics against Bacterial Pathogens” to discuss details and critical parameters of various PK/PD methods and identify approaches for linking human pharmacokinetic (PK) data and drug efficacy analyses. The workshop participants included individuals from academia, industry, and government.

## INTRODUCTION

Prior decades were characterized by the introduction of an abundance of novel antibacterial agents. Developers of these agents leveraged large studies yielding multiple indications. Noninferiority trials comparing a test agent to a standard of care comparator was the typical approach to registration. With the progressive emergence of antimicrobial resistance, recognized as a major threat to both the public and to medical progress, the scientific and regulatory community began to think differently about the requirements of clinical data to support new agents aimed at treating serious and life-threatening infections caused by highly resistant pathogens (1). The conduct of clinical trials to demonstrate efficacy against drug-resistant bacterial species is challenging, mainly because of the lack of sufficient patients who are infected with target bacterial species. Thus, it is important to consider how other information like pharmacokinetics/pharmacodynamics (PK/PD) can support the clinical effectiveness of new antibacterial drugs. Over the past 5 years, both the United States Food and Drug Administration (U.S. FDA) (2) and the European Medicines Agency (EMA) (3, 4) issued guidance documents enabling streamlined development programs with the caveat that agents should be used only in the setting of limited therapeutic options. While some differences exist between the agency guidance documents, one area of alignment between the U.S. FDA and EMA is that robust PK/PD data are central to antibacterial drug development programs, although the exact scope of such data requirements is loosely defined.

In the current antibiotic development era, many different sources of PK/PD data are integrated during the drug development process to support dose selection and to provide a measure of certainty ahead of larger clinical trials. Successful application of such approaches, together with integration of clinical PK/PD data, is now used to provide a more informative drug development package to support the approval of new antibiotics. There are now proposals to conduct more focused clinical trials and to require robust PK/PD data packages when a development program for an antibacterial agent for the treatment of infections arising from rare pathogens can be supported by only limited clinical data (1). Whether for the treatment of infections arising from pathogens with usual drug resistance or those arising from multidrug-resistant or extensively drug-resistant pathogens, the assessment of clinical PK/PD using data from the target patient population is useful. Here, we focus on considerations for clinical PK/PD analyses and dose selection and the importance of assessing drug penetration in the lung for the treatment of pneumonia, with an emphasis on special populations, including patients with renal impairment (RI) and augmented renal function, as well as on dosing in obese and pediatric patients. Robust clinical PK/PD analyses also require an accompanying robust nonclinical PK/PD package. A robust nonclinical PK/PD package is one that provides PK/PD targets for efficacy that are informed by data from two or more experimental systems, including one-compartment *in vitro* and *in vivo* infection models. PK/PD targets should be based on data from a relevant collection of isolates for which the MIC range and resistance mechanisms encompass those expected to be encountered clinically. The sample size of such isolate collections should be sufficient to characterize variability in the magnitude of PK/PD targets for efficacy. Lastly, such data should be externally consistent and reproducible and data for selected isolates should thus be based on experiments conducted by two or more groups of investigators. Given the importance of ensuring the durability of the antibacterial dosing regimen, studies using static *in vitro* systems should be undertaken to characterize mutation frequency and to determine MIC values for mutant isolates. Selected dosing regimens should be pressure tested for the ability to suppress amplification of resistant bacterial subpopulations. The *in vitro* hollow fiber infection model, which allows studies of longer durations, is the most common infection model used to evaluate resistance amplification. Inclusion of such data increases the robustness of the nonclinical PK/PD package. In June 2017, The National Institute of Allergy and Infectious Diseases, part of the National Institutes of Health, organized a workshop entitled “Pharmacokinetics-Pharmacodynamics (PK/PD) for Development of Therapeutics against Bacterial Pathogens” to discuss details and critical parameters of various PK/PD methods and to identify approaches for linking human pharmacokinetic (PK) data and drug efficacy analyses. The workshop participants included individuals from academia, industry, and government, including the United States Food and Drug Administration (U.S. FDA). This and the accompanying minireview on nonclinical PK/PD (5) summarize the workshop discussions and recommendations, which are the opinions of individual participants and are not meant to serve as regulatory guidance.

## LEVERAGING PHARMACOMETRICS FOR DOSE SELECTION

PK/PD modeling and simulation approaches typically fall into three main categories and have been utilized to various degrees in support of recent drug approval of antibacterial small-molecule new molecular entities (NMEs) (6, 7). Table 1 shows a list of entities for which population PK (PopPK) analysis, exposure-response (E-R) analysis, and probability of target attainment (PTA) analysis have been applied to the drug development programs since 2009.

**TABLE 1:** FDA-approved antibacterial small-molecule new molecular entities between 2009 and 2015^*a*^

Yr	Drug name	Pharmacometric analysis	Indication
2009	Telavancin	PopPK	cSSSI, HABP/VABP
	Besifloxacin	NA	Bacterial conjunctivitis

2010	Ceftaroline fosamil	PopPK, E-R, PTA	ABSSSI, CABP

2011	Fidaxomicin	NA	Clostridium difficile-associated diarrhea

2012	Bedaquiline	PopPK, E-R	Combination therapy for MDR-TB

2014	Dalbavancin	PopPK, E-R	ABSSSI
	Oritavancin	PopPK, E-R, PTA	ABSSSI
	Tedizolid phosphate	PopPK, E-R, PTA	ABSSSI
	Ceftolozane and tazobactam	PopPK, PTA	cIAI, cUTI

2015	Ceftazidime and avibactam	PopPK, E-R, PTA	cIAI, cUTI

a PopPK, population pharmacokinetic; E-R, exposure-response; PTA, probability of target attainment; NA, not applicable due to local antibacterial treatment; cSSSI, complicated skin and skin structure infections; HABP/VABP, hospital-acquired bacterial pneumonia/ventilator-associated bacterial pneumonia; ABSSSI, antibacterial skin and skin structure infections; CABP, community-acquired bacterial pneumonia; MDR TB, multidrug-resistant tuberculosis; cIAI, complicated intraabdominal infections; cUTI, complicated urinary tract infections.

PopPK analysis is a well-accepted pharmacometrics methodology to predict the PK characteristics of drugs in patients. PopPK analysis can provide the exposure information used as an input to E-R and PTA analyses. The covariate analysis within a PopPK model evaluates the impact of demographic parameters on exposure and determines the need for dose adjustment in specific populations, such as obese patients, geriatric patients, or patients with renal/hepatic impairment. The robustness of the PopPK model is dependent upon the quality and quantity of PK data included in the model together with the associated demographic data from subjects contributing PK data. PK and its variability can differ from indication to indication, as well as between healthy subjects and infected patients. Ideally, PopPK analysis used to inform E-R or PTA analyses should include sufficient PK data from patient populations with the target indication(s), with the PK/PD target determined using appropriate preclinical infection models.

E-R analysis evaluates the relationship between drug exposure and outcomes. The exposure can be characterized as the dose, area under the concentration versus time curve (AUC), maximum concentration (*C*_max_), or minimum concentration (*C*_min_), while the response can represent clinical outcomes such as safety, efficacy, or a biomarker of interest. E-R analysis plays a key role in dose selection through all phases of drug development. It should be noted that the value of dose as a metric of drug exposure can be limited, as examples of clinical trials with a sufficiently broad dose range to establish such a relationship are increasingly rare due to both advances in PK/PD to support dose optimization and ethical considerations for administering suboptimal doses in this patient population. Further, the use of dose ignores the variability between patients in exposure to drug as captured in pharmacokinetic parameter values. Due to these limitations, here we focus on drug exposure measures for establishing E-R relationships.

For antibacterial agents, E-R analyses for efficacy are typically conducted by utilizing the PK/PD indices (e.g., free-drug [*f*] area under the concentration-time curve [AUC]/MIC [*f*AUC/MIC], free-drug maximum concentration [*f*C_max_/MIC], and percentage of the dosing interval during which free drug concentrations are above the MIC [%*fT*>MIC]) which represent measures of unbound exposure indexed to the organism susceptibility (as represented by the MIC). PK/PD indices evaluated for E-R analyses are typically those identified to most closely associate with efficacy based on nonclinical PK/PD studies. Of the 10 recent small-molecule antibacterial NME applications, 6 included an E-R analysis. E-R analysis for efficacy endpoints may not be informative for some antibacterial NMEs because phase 2 and 3 trials often do not include a wide enough range of exposures or a sufficient number of treatment failures due to optimal dose selection decisions based on the use of preclinical PK/PD data, phase 1 data, and Monte Carlo simulation.

In general, lack of identification of an E-R relationship for efficacy is expected in evaluating data from patients treated with a PK/PD optimized dosing regimen. In cases where a relationship is identified, this is typically based on determination of an optimal threshold value for the PK/PD indices, which are treated as dichotomized variables. Thresholds may be determined using the first split of a classification or regression tree or a receiver operating characteristic curve or may be based on a model-predicted threshold for achieving a target response. Relationships based on dichotomized variables for PK/PD indices allow patients with both lower PK/PD indices and lower percentages of successful response to be distinguished from those with higher PK/PD indices and higher percentages of successful response (8). When PK/PD relationships based on clinical data are not found, assessments of distributions of PK/PD indices achieved relative to nonclinical PK/PD targets for efficacy represent a useful assessment to confirm the original basis for dose selection.

PTA analysis is an assessment of the probability of attaining a PK/PD target in a patient population with a specific dosing regimen. The PK/PD target is determined from preclinical studies (9, 10) or may be determined from the clinical data in cases where an E-R relationship is identified, as discussed above. It is a tool to support dose selection to evaluate whether a given dose would be effective in specific patient populations or against a specific organism. PTA analysis was included in 5 of 10 antibacterial NME applications as an essential component by integrating the information from PopPK analyses in healthy volunteers and/or patients with the PK/PD target determined from *in vitro* microbiological studies and *in vivo* animal infection studies.

There is also the potential to leverage physiologically based pharmacokinetic (PBPK) analyses to predict the effects of intrinsic and extrinsic factors on drug exposure to support dosing recommendations in specific clinical situations. Although PBPK analysis has not been included in any of 10 antibacterial small-molecule NME submissions, it is increasingly used in other therapeutic areas during the assessment of drug-drug interactions and dose individualization in subpopulations. However, a key consideration for PBPK analysis for antibacterial agents is that such models rely on estimations of physiological parameters, including organ blood flow, derived from the physiology literature. Such blood flow can be significantly altered in patients with sepsis or the critically ill, which may limit the utility of the PBPK approach in the absence of robust physiological and PK data in the patient population to appropriately tune such models. A document providing FDA guidance regarding format and content of PBPK analysis became publicly available in December of 2016 to facilitate the incorporation of this analysis tool into NME submissions to support decision-making during drug development (11). For antibacterial agents, PBPK should be considered only in circumstances in which physiological parameters in the target patient population during acute infection conditions are available.

## CLINICAL PK/PD DATA CONSIDERATIONS

**FIG 1:**
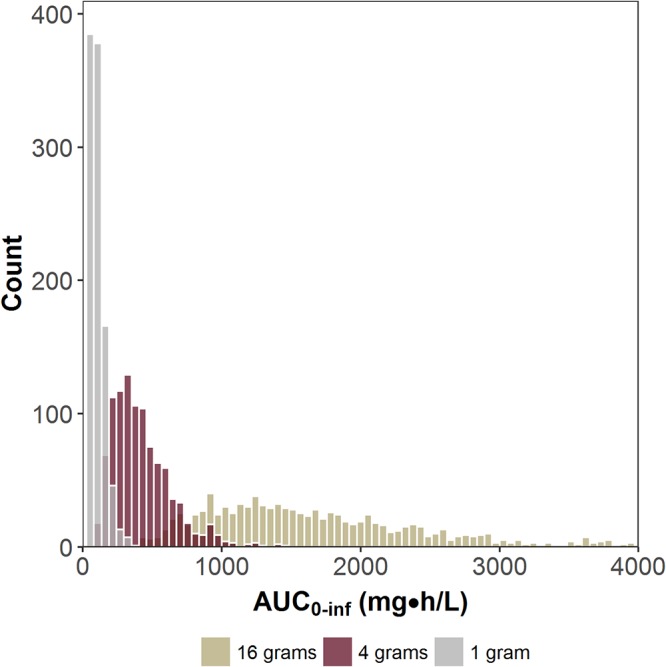
Area under the plasma concentration-time curve (AUC_0–inf_) distributions for three different doses of a hypothetical antibacterial agent. (Reprinted from reference 8 with permission from Elsevier.)

Phase 2 studies can be conducted to evaluate the efficacy and safety of two or more dosing regimens. However, the value of typical phase 2 study designs, which involve the study of doses similar in magnitude, needs to be considered in the context of the current paradigm for developing antibacterial agents. As demonstrated by Fig. 1, values representing AUC from 0 to infinity (AUC_0–inf_) after single doses of 1, 4, and 16 g of a hypothetical antibacterial agent would have to be many folds apart in order to avoid overlap of distributions (8). Given the concerns about administering a low dose whereby a number of patients would have drug exposures that approach zero, the evaluation of dosing regimens with minimally overlapping AUC distributions is not feasible. With the increased confidence that comes from using preclinical PK/PD and phase 1 PK data to support dose selection, phase 2 dose-ranging studies to discriminate efficacy between two dosing regimens that have overlapping distributions of drug exposures may be less useful.

Unless there are safety concerns or uncertainties about PK/PD predictions, it may be possible to carry out a more streamlined development program, conducting phase 1b studies or focused phase 2 or adaptive design clinical trials prior to studying a PK/PD optimized regimen in phase 3 studies. With this approach, a PK/PD-optimized regimen could be chosen for direct evaluation in a phase 3 randomized-controlled trial, streamlining the drug development process. However, for such an approach to be successful, it will be important to study PK in the target patient population through the execution of a phase 1b study and using phase 1 PK data from special populations that allow quantification of covariates of PK (e.g., healthy volunteers with renal impairment for drugs that are renally cleared). While inflation of the variance structure of the PK parameters of healthy subjects from phase 1 studies is a useful strategy to estimate the PK in infected patients, it will still be important to conduct phase 1b or 2 studies to evaluate PK in the target population and to confirm assumptions about dose selection prior to initiating phase 3 studies. If a phase 2 study is conducted, E-R analyses for both efficacy and safety endpoints should be investigated prior to the initiation of phase 3 studies and such data should later be pooled with phase 3 data to further enrich the sample size of evaluable patients.

Although it is often impossible to assess the effects of various doses of a new treatment because it is not ethical to knowingly “underdose” patients, such data, when available, are informative. The value of phase 2 data to assess dosing regimens and duration and safety using a pharmacometric approach can be illustrated using the example of brilacidin-treated patients with acute bacterial skin and skin structure infections (ABSSSI) who were enrolled from two phase 2 studies (12). Brilacidin is a defensin mimetic that disrupts cell membrane integrity and has activity against Gram-positive and Gram-negative organisms, including methicillin-resistant Staphylococcus aureus. Pooled data from the two phase 2 studies, the first of which provided active drug for 5 days and the second of which provided active drug for 1 or 3 days, allowed the formation of a rich data set that consisted of six different dose levels and three different therapy durations. The second study, which provided the benefit of increasing the sample size of the analysis population, was initiated to evaluate a loading dose and a shorter duration of therapy. E-R relationships were explored for efficacy endpoints assessed early in therapy and at traditional time points, at the end-of-therapy (EOT) or test-of-cure (TOC)/short-term follow-up (STFU) visits. Relationships between brilacidin exposure and two safety endpoints, systolic blood pressure and numbness/tingling, were assessed. E-R relationships for ≥20% reductions from baseline in lesion area on day 2 and clinical success at EOT and TOC/STFU and each of the two latter safety endpoints were identified (12). The application of these E-R relationships to simulated data generated using a PopPK model was carried out with the objective of discriminating among candidate brilacidin dosing regimens (13). As illustrated by this example, E-R relationships for efficacy and safety, when identified, can be used to assess risk versus benefit and the value proposition for further clinical development. Carried out in late-stage development, PK/PD analyses for efficacy and safety using phase 3 data produce results that can be used to support the identification of susceptibility breakpoints and patient populations with increased risk of failure and/or safety events. Such data can then be used to inform labeling and/or clinical practice guidelines.

The identification of PK/PD relationships for efficacy and safety based on clinical data collected during development has the potential to inform dosing practices postapproval, especially in the landscape of shifting MIC values. In the example of daptomycin, which was studied in patients with Staphylococcus aureus bacteremia with or without infective endocarditis enrolled in a phase 3 study (14), population PK and PK/PD analyses of efficacy and safety were undertaken to support the supplemental new drug application (sNDA) for this indication (15, 16). Pharmacokinetic samples were obtained from 106 patients, and a PopPK model was developed (15). This model allowed the evaluation of E-R relationships between measures of drug exposure normalized to MIC and (i) clinical outcome, (ii) toxicity (serum creatine phosphokinase [CPK] elevation), and (iii) resistance emergence during therapy with daptomycin (15, 16). While the results of these analyses were used to support the sNDA, the application of these E-R relationships was also useful to evaluate higher daily doses of daptomycin (8 or 10 mg/kg of body weight/day) relative to the approved 6 mg/kg/day dosing regimen in this patient population postapproval (16). Using the PopPK model, the three E-R relationships described above, and Monte Carlo simulation, the likelihood of a good outcome for all three endpoints was determined for each dosing regimen. The results of these analyses failed to demonstrate large increases in the percentages of simulated patients who achieved clinical success or large reductions in the percentages of patients with decreased susceptibility with dose increases in daptomycin from 6 to 10 mg/kg/day. However, percent probabilities of clinical success that had increased by 10% were demonstrated among subgroups of simulated patients defined by selected comorbidities who received 10 mg/kg/day relative those receiving 6 mg/kg/day. Although the percent probability of CPK elevation doubled over this dose range (7.3 to 15.6%), clinicians need to weigh such risks in the context of the mortality and severe morbidity associated with serious staphylococcal infections. These data served to provide guidance to clinicians with regard to the probabilities of clinical success and resistance emergence against the probability of toxicity, thereby providing data for the assessment of risk versus benefit. The examples described above demonstrate the value of data from E-R analyses of clinical trial results during clinical development and the application of such data postapproval to further assess dose.

## PATIENTS WITH PNEUMONIA

In assessing the PK/PD of an antibiotic, it is critical to consider the concentrations achieved at the site of infection (17–19). While free drug concentrations in plasma are often viewed as representing an acceptable approximation for free drug concentrations at the site of infection, this is not always the case. This is of concern in the treatment of pneumonia and concentrations of drug in the epithelial lining fluid (ELF). The drug concentrations in ELF are typically measured in clinical studies from samples obtained via bronchoalveolar lavage (BAL), an invasive process that is generally limited to a single concentration time point per patient. Historically, analysis of ELF drug penetration data was limited to obtaining ratios of drug concentrations in the ELF to those determined simultaneously in plasma. This is a flawed approach as the plasma-to-ELF penetration ratio can change as a function of time due to system hysteresis. PopPK modeling is used to estimate area under the concentration-time curve for epithelial lining fluid (AUC_ELF_) with limited PK samples because of its ability to estimate PopPK and their associated dispersions for subjects with minimal sampling times. Once the PopPK in ELF are established, Monte Carlo simulation can then be used to estimate the ability of a drug to penetrate the site of infection, defined as the AUC_ELF_/AUC_plasma_ ratio, and to characterize its ability to achieve the desired PK/PD target at that site (20, 21).

Prior to conducting clinical trials, obtaining ELF penetration data in healthy volunteers is necessary to ensure appropriate dosing in terms of attaining the PK/PD target at the infection site. In these assessments, it is typically assumed that all measured drug in the ELF is unbound (free) and that protein binding in the ELF is negligible (18). However, the assumption that protein binding in ELF is negligible has not been validated and requires further assessment. In point of fact, proteins have been measured in ELF using bronchoalveolar lavage fluid for decades (22). It is straightforward to correct the measured concentrations for urea dilution. Binding could be estimated for the agent of interest employing this concentration of binding protein (most often albumin). While straightforward, this has yet to be performed for any modern antibacterial agent.

**FIG 2:**
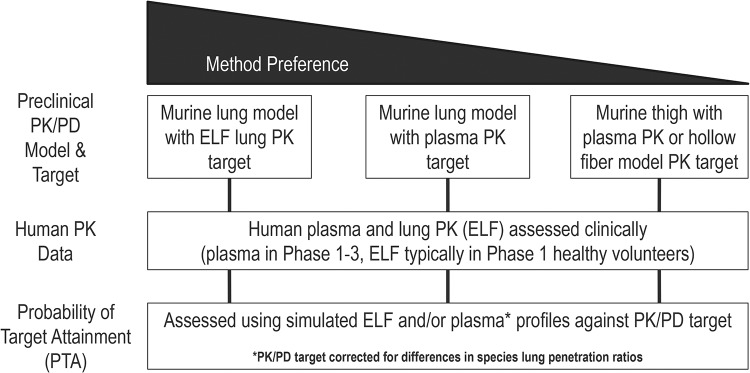
Approaches for assessing optimal dosing for the treatment of pneumonia.

The preferred method for dose selection for antibacterial agents for the treatment of patients with pneumonia is to assess the probability of PK/PD target attainment using preclinical ELF PK/PD targets from neutropenic murine infection models and simulations of ELF concentration-time profiles. However, as shown in Fig. 2, alternative approaches for pneumonia dose selection have been pursued. Each of these methods has advantages and disadvantages. Leveraging the murine lung infection model for PK/PD target derivation ensures that the target comes from an *in vivo* system where infection is established at the same site of infection (the lung) as the intended indication in humans. Simulations of human ELF concentration-time profiles provide the most nearly proximal assessment of free-drug exposures to the infection site. But there remain questions as to how well the PK variability is captured in these data, given the limitations of current ELF sampling study designs. More specifically, variability may be overestimated in the ELF data due to BAL fluid sampling, urea correction, and other methodological sources, as opposed to being a representation of the true biological variability. To address or circumvent these issues with ELF variability and to leverage the measured patient plasma PK variability captured in phase 2 and 3 data, PTA analysis can be conducted using plasma PK from both murine lung and human subjects, with a correction made for cross-species differences in lung penetration ratios (Fig. 2). As described above, ELF data are typically collected in healthy volunteers, and levels of lung penetration may differ between healthy volunteers and patients, due to inflammation and other factors. Despite these concerns, ensuring optimal drug exposures at the infection site remains of paramount importance for antibacterial dose selection, especially for patients with pneumonia, for which drug concentrations in the lung can be assessed both in preclinical models and in clinical studies.

## DOSE SELECTION CONSIDERATIONS IN SPECIFIC POPULATIONS

It is important to recognize that overall PTA analyses provide an expectation of efficacy across all patient populations. For antibiotics where there is no clinically significant relationship between PK parameters and patient covariates, this is not an issue. When PK parameters (e.g., volume of distribution and clearance) vary as a function of well-defined patient covariates, it is important to assess the PTA profiles across all important patient populations and determine if dosage adjustments are required for them (23). The populations where these considerations are typically applied and the points to be considered are described in the following sections.

### Patients with renal impairment.

In the United States and Europe, specific guidance and criteria for PK analyses to promote optimal dosing in patients with renal impairment are available. The Cockcroft-Gault and modification of diet in renal disease equations are considered suitable options to characterize the renal function of patients for the purpose of drug dose adjustment in adults with renal impairment (24–26). Note that each of these methods of estimating renal function was originally designed to be used in the setting of chronic kidney disease and may not be appropriate to estimate renal function in patients with acute renal impairment as they rely on a single-point estimate of serum creatinine. These equations require a fundamental expectation of homeostasis, which is often not the case in acutely ill patients (27).

The phase 3 noninferiority trials focusing on complicated intra-abdominal infections (cIAI) that compared ceftazidime-avibactam (CAZ-AVI) with metronidazole to meropenem (RECLAIM 1 and 2 trials) is one of the notable examples in which underdosing based on the Cockcroft-Gault creatinine clearance (CL_CR_) equation may have resulted in discordant response rates between treatment arms. In the RECLAIM trials, clinical cure rates were lower in the CAZ-AVI plus metronidazole group than in the meropenem-treated group among patients with moderate renal impairment (MRI) at baseline (28). On the basis of these observations, the CAZ-AVI prescribing information includes a warning regarding decreased efficacy in patients with moderate renal impairment (CL_CR_ 30 to 50 ml/min) (29). Note that similar results (i.e., decreased efficacy in patients with renal impairment) were observed in phase 3 trials of other antibiotics such as telavancin and ceftolozane-tazobactam (29, 30).

Monte Carlo simulations performed prior to RECLAIM 1 and 2 indicated that the CAZ-AVI dose selected for patients with moderate renal impairment had a highly favorable (>90%) joint PTA profile (50% *fT*>MIC for ceftazidime and 50% *fT*>*C_T_* [threshold concentration] of 1 mg/liter for avibactam) for patients with infections with CAZ-AVI MIC values of ≤16/4 mg/liter. While the moderate renal impairment dose regimen was found to have a favorable joint PTA profile, nearly 70% of study patients with a baseline CL_CR_ level of <50 ml/min in RECLAIM 1 and 2 experienced an improvement of renal function to >50 ml/min within 72 h of study drug dosing initiation (31). Therefore, the potential of underdosing due to the absence of an immediate dose increase in the setting of improved renal function may have resulted in deleterious patient outcomes with CAZ-AVI due to suboptimal drug exposure in this specific subpopulation.

**FIG 3:**
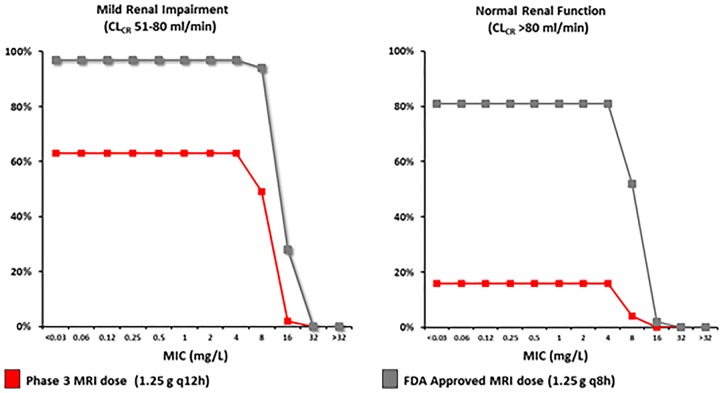
Probability of target attainment for the FDA-approved moderate renal impairment ceftazidime-avibactam dose relative to the phase 3 clinical trial dose among patients whose renal function improved from moderate impairment to the mild impairment (CL_CR_, 51 to 80 ml/min) and normal function (CL_CR_, >80 ml/min) categories. (Reprinted from reference 32 with permission from John Wiley & Sons, Inc.)

In RECLAIM 1 and 2, patients with moderate renal impairment (MRI) (CL_CR_ 30 to 50 ml/min) received a 66% total daily dose reduction for CAZ-AVI (2.5 g intravenous every 8 h to 1.25 g every 12 h). As shown in Fig. 3 (32), the PTA is approximately 60% for the MRI dose among patients whose renal function improves to the mild renal impairment range. Among patients whose renal function improves to the normal range, the PTA drops to less than 20% for the MRI dose. To mitigate the potential for this underdosing, the recommended dose of CAZ-AVI for patients with moderate renal impairment was increased from 1.25 g every 12 h to 1.25 g every 8 h. The PTA with 1.25 g every 8 h is greater than 95% for patients with mild renal impairment and ∼80% for patients with normal renal function. Furthermore, this updated MRI dosing scheme did not result in excess accumulation, as measured by the AUC at steady state (Table 2) (33).

**TABLE 2:** Daily CAZ-AVI exposure for the approved CAZ-AVI moderate renal impairment dose of 1.25 g every 8 h in patients with normal renal function or mild or moderate renal impairment^*a*^

Renal function (CL_cr_ range, ml/min)	CAZ-AVI dose (g q8h)	AUC_0–24,ss_ (mg·h/liter), median (CV%)	
		CAZ	AVI
Normal (>80)	2.5	91.2 (23)	518 (30)
Mild (>50 to 80)	2.5	126 (28)	783 (31)
Moderate (>30 to 50)	1.25	116 (28)	640 (31)

a CAZ-AVI, ceftazidime-avibactam; q8h, every 8 h; AUC_0–24,ss_, area under the plasma concentration-time curve at steady state from 0 to 24 h; CV%, percent coefficient of variation.

Rather than relying on CL_CR_ or GFR estimation equations to determine appropriate dosing regimens in renal insufficiency, there are alternative equations that can potentially be used to more accurately characterize renal function in the setting of rapidly changing serum creatinine levels (27, 28, 34). In contrast to relying on a point estimate of the serum creatinine to estimate renal function, these equations quantify renal function by considering the magnitude with which the serum creatinine level is increased or decreased compared to its steady-state value and the rapidity of the change; however, these approaches have not been validated to guide drug dosing. Future antibiotic development should consider the evaluation and validation of these approaches to estimate renal function for determining optimal drug dosing in patients with rapidly changing renal function.

### Patients with augmented renal function.

The need for appropriate dose modifications for patients with renal impairment also applies in the opposite direction for patients with augmented renal clearance (ARC). ARC, often defined as a CL_CR_ level of >130 ml/min, is being increasingly described in subsets of critically ill patients. It is estimated that approximately 30% to 65% of patients in the intensive care unit (ICU) have ARC despite the presence of a normal serum creatinine concentration (35, 36). Patient populations with the highest reported incidence of ARC include those with major trauma, sepsis, traumatic brain injury, subarachnoid hemorrhage, and central nervous system infections. Critically ill trauma patients are often hypermetabolic and frequently require aggressive fluid resuscitation. This may result in increased renal clearance of drugs and higher volumes of distribution (37, 38). Published data suggest these patients often require more intensive dosing schemes for antibiotics that are eliminated mainly by the kidneys, due to their altered physiology. It is also important to note that augmented renal function can be associated with alterations in a number of other physiologic processes that may affect the antibiotic exposure profile. Compensatory nonrenal elimination via the gut or hepatic system may be stimulated, potentially resulting in enhanced drug clearance (20, 39). Furthermore, patients with sepsis or septic shock may have enhanced clearance of drugs caused by increasing cardiac output, leading to higher blood flow to all clearance organs (40). This phenomenon has been increasingly reported and indicates that dose supplementation may be required in patients with ARC (35). The relevance of these findings is underscored by a recent multicenter study by Roberts et al. (41) which found that ICU patients receiving β-lactams who failed to achieve critical PK/PD targets were more likely to experience negative outcomes than those who achieved PK/PD targets.

Similar to efforts to identify patients with rapidly improving renal function, estimated CL_CR_ or glomerular filtration rate (GFR) equations that rely on serum creatinine concentrations do not accurately identify patients who exhibit ARC. Collecting 8-h continuous urine is recommended in patients at high risk for ARC to assess CL_CR_ versus empirical CL_CR_/GFR estimation equations. Alternatively, therapeutic drug monitoring (TDM) or use of GFR clearance biomarkers could be considered, although best practices for TDM merit further consideration (35).

### Appropriate extrapolation of dose for body size.

When selecting an antibiotic dosing regimen, consideration should be given to whether dosing should be fixed or weight based (42). Antibiotic dosing based on body surface area (BSA) scaling is not frequently conducted in adult patients. For weight-based dosing, the assumptions are that key PK parameters (i.e., clearance and volume of distribution) change proportionately with weight and that weight-based dosing is necessary to achieve isometric exposure distributions across the continuum of weights. Conversely, the lack of association between weight and key PK parameters permits use of a fixed dosing regimen as it is likely to result in comparable exposures across the weight continuum (42).

Early clinical studies typically included adults within a narrow range of body size, hindering the ability to fully evaluate the association between weight and key PK parameters across the current weight distribution in the United States (43). It is now estimated more than one-third of adults in the United States are obese, defined as a body mass index (BMI) of ≥30 kg/m^2^ (44). Therefore, early-phase clinical trials should enroll patients across the entire weight continuum to permit appropriate dose extrapolation for body size. As part of these evaluations, alternate body size descriptions such as BSA, BMI, ideal body weight, adjusted body weight, and lean body weight should be considered as alternative measures of body size in the PK analyses. This will help ensure that the dosing strategy (i.e., fixed versus body size descriptor base) selected will result in isometric exposures across the distribution of weights observed in clinical practice (43).

### Pediatric patients.

Full extrapolation of efficacy data from adults to pediatrics may be appropriate if it is reasonable to assume that the two populations have (i) similar disease progressions, (ii) similar responses to intervention, and (iii) similar exposure responses. A decision tree illustrating the use of an E-R relationship for bridging efficacy data in an adult population to a pediatric population is presented in the FDA draft guidance for industry (45).

Full extrapolation for efficacy is applicable for many antibacterial products. It is only when efficacy in pediatric patients can be fully extrapolated from adult studies that pediatric PK and safety studies are solely required to establish the right dose. Establishing the pediatric dose can be performed by exposure matching to adults in the case of full extrapolation, and the same occasionally applies to partial extrapolation. For antibacterial drugs, *a priori* standards for exposure matching include (i) identification of the target PK/PD index metric (e.g., AUC/MIC, *C*_max_/MIC, and/or %*T*>MIC); (ii) the specific target value or range of this metric; and (iii) overlapping an acceptable percentage of the adult exposure distribution.

Care should be taken to characterize and understand when differences in pharmacokinetics (beyond what can be described by allometric scaling) may manifest for antibacterial agents, especially in very young patients. Enzyme and clearance organ maturation differences may have a significant impact on drug PK, and the maturation of various elimination processes can occur over a range of the first weeks to years of life. As PK data are collected in pediatric subjects below 2 years of age, analyses should specifically look for evidence of nonlinearities in drug clearance due to maturation of elimination pathways. From a PK/PD perspective, changes in drug clearance (and half-life) has also been reported to potentially result in PK/PD driver “switching,” where if the half-life is substantially extended, the PK/PD driver can switch from being time driven (%*T*>MIC or *C*_min_/MIC) to being concentration driven (AUC/MIC) (46, 47). This can be accounted for either through the examination of PK/PD relationships in an *in vitro* infection model where half-lives can be easily adjusted or through pharmacometric approaches where the entire time courses of both PK and PD data are modeled (46, 47).

## CONCLUSIONS

Pharmacometrics represents an embraced set of tools that allow antibacterial agents to be developed in a streamlined and efficient manner. The assessment of dosing regimens for antibacterial agents utilizes a well-accepted paradigm that includes using robust preclinical PK/PD data and clinical PK data to select a candidate dosing regimen that has a high probability of achieving the PK/PD targets associated with outcomes of interest. Studies have shown that the use of such analyses to guide the dose selection process increases the probability of a successful NDA (48) and, more importantly, ensures that patients are adequately treated for severe and potentially fatal infections. Future challenges in this area include the need to better understand, characterize, and predict PK profiles across the populations likely to be encountered in clinical practice. At both extremes of renal function, it is critical for optimal dose selection to study drug clearance and exposure profiles prior to initiating phase 3 studies. Similarly, efforts should be made to ensure adequate dosing across the entire weight continuum for the target patient population. It is also important to conduct appropriate studies and analyses for proper dose selection in pediatric patients with minimal delay beyond introduction to adults.

To accomplish the goals described above, it will be important to ensure that robust clinical PK and PK/PD data packages are assembled during early- and late-stage development (1, 8). During early-stage development, the phase 1 PK component of this package will need to include data from studies that characterize covariates describing the PK of the antibacterial agent (e.g., renal and/or hepatic impairment studies). Also, if relevant, PK studies need to be conducted to collect data about effect site exposure (i.e., evaluating ELF PK for pneumonia indications). Finally, as described here, inclusion of data from phase 1b studies will allow the characterization of PK and the associated variability of such PK in the target patient population. In late-stage development, PK data are needed from all patients enrolled in pivotal trials. Evaluation of such clinical PK/PD data will allow the confirmation of adequate drug exposure and the evaluation of potential E-R relationships for efficacy and safety endpoints. This final step will allow confirmation of dose selection decisions made during earlier stages of development and will enable patient populations with increased risk of failure and/or safety events to be identified. The conduct of appropriate studies and analyses to support dose selection in pediatric patients, with appropriate bridging to preclinical PK/PD and clinical PK and PK/PD data packages, will ensure minimal delay beyond introduction to adults for the availability of such agents for pediatric patients. As development paths for indications involving multidrug-resistant or extensively drug-resistant pathogens evolve, it will be even more important to ensure that the preclinical PK/PD packages that were described earlier and in the companion paper by Bulitta et al. (5) and the clinical PK/PD data packages are strategically designed to account for the limited clinical data collected. In conclusion, consideration of the studies and analyses described here to support dose selection decisions for antibacterial agents will reduce the likelihood of drug development failures and, more importantly, result in approved dosing regimens associated with optimized patient outcomes.
